# Aripiprazole Lauroxil, a Novel Injectable Long-Acting Antipsychotic Treatment for Adults with Schizophrenia: A Comprehensive Review

**DOI:** 10.3390/neurolint13030029

**Published:** 2021-07-01

**Authors:** Kunal Maini, Haley Gould, Jessica Hicks, Fatima Iqbal, James Patterson, Amber N. Edinoff, Elyse M. Cornett, Adam M. Kaye, Omar Viswanath, Ivan Urits, Alan D. Kaye

**Affiliations:** 1 Department of Psychiatry, Louisiana State University Shreveport, Shreveport, LA 71103, USA; kmaini@lsuhsc.edu (K.M.); JPatte@lsuhsc.edu (J.P.II); 2 Shreveport School of Medicine, Louisiana State University, Shreveport, LA 71103, USA; hgoul1@lsuhsc.edu (H.G.); jhick6@lsuhsc.edu (J.H.); fiqba2@lsuhsc.edu (F.I.); 3 Department of Anesthesiology, Louisiana State University Shreveport, Shreveport, LA 71103, USA; ecorne@lsuhsc.edu (E.M.C.); ivanurits@gmail.com (I.U.); alan.kaye@lsuhs.edu (A.D.K.); 4 Department of Pharmacy Practice, Thomas J. Long School of Pharmacy and Health Sciences, University of the Pacific, Stockton, CA 95211, USA; akaye@PACIFIC.EDU; 5 College of Medicine-Phoenix, University of Arizona, Phoenix, AZ 85004, USA; viswanoy@gmail.com; 6 Department of Anesthesiology, Creighton University School of Medicine, Omaha, NE 68124, USA; 7 Valley Anesthesiology and Pain Consultants—Envision Physician Services, Phoenix, AZ 85004, USA; 8 Beth Israel Deaconess Medical Center, Department of Anesthesiology, Critical Care, and Pain Medicine, Harvard Medical School, Boston, MA 02215, USA

**Keywords:** schizophrenia, schizophrenia treatment, aripiprazole lauroxil, long-acting injections, aristada

## Abstract

Purpose of Review. This is a comprehensive review of the literature regarding the use of Aripiprazole lauroxil for schizophrenia. This review presents the background, evidence, and indications for using aripiprazole lauroxil to treat schizophrenia in the context of current theories on the development of schizophrenia. Recent Findings. Schizophrenia is a chronic mental health disorder that currently affects approximately 3.3 million people in the United States. Its symptoms, which must be present for more than six months, are comprised of disorganized behavior and speech, a diminished capacity to comprehend reality, hearing voices unheard by others, seeing things unseen by others, delusions, decreased social commitment, and decreased motivation. The majority of these symptoms can be managed with antipsychotic medication. Aripiprazole lauroxil is a long-acting intramuscular injection that works as a combination of partial agonist activity at D_2_ and 5-HT_1A_ receptors combined with antagonist activity at 5-HT_2A_ receptors. It can be dosed as a 4-, 6-, or 8-week injection, depending on oral dosage. Aripiprazole lauroxil was FDA approved in October of 2015. Summary. Schizophrenia is a severe psychiatric disorder if left untreated. There are multiple medications to help treat schizophrenia. One antipsychotic agent, aripiprazole lauroxil, offers long duration injections that optimize and improve compliance. Known side effects include weight gain, akathisia, neuroleptic malignant syndrome, tardive dyskinesia, and orthostatic hypotension. Aripiprazole lauroxil is an FDA-approved drug that can be administered monthly, every six weeks, or every two months and has been shown to be both safe and effective.

## 1. Introduction

Schizophrenia is a psychotic disorder that can be characterized as having positive symptoms, negative symptoms, and cognitive dysfunction. Positive symptoms consist of the presence of behaviors or thoughts that are not typically present, such as psychosis, delusions, hallucinations, disorganized speech, and behavior [1]. Negative symptoms are characterized by social withdrawal, flat affect, anhedonia, and low energy [1]. The positive and negative symptoms of schizophrenia are thought to be a result of dopaminergic dysregulation. Cognitive impairment is also a main component in schizophrenia and is largely refractory to treatment [2]. Cognitive deficits include impairment in attention, working memory, episodic memory, processing speed, and executive function [3]. A decline in social and cognitive functions generally presents itself > 10 years before the onset of psychotic symptoms and leads to those with schizophrenia encountering problems with their ability to maintain social relationships, employment, and independence [3,4]. Although treatment with antipsychotics and psychosocial interventions can help increase quality of life, the severity of negative symptoms and cognitive impairment at onset can be a determining factor in treatment efficacy [5]. The Diagnostic and Statistical Manual of Mental Disorders 5th edition requires that psychotic symptoms be present for over six months [6]. The lifetime prevalence of major depression in schizophrenia was found to be between 30–50% [7,8]. 34% of patients were found to have a lifetime diagnosis of alcohol abuse or dependence [9].

The treatment of schizophrenia is a combination of antipsychotic medications and nonpharmacological treatment [10]. The combination of pharmacological and nonpharmacological interventions can result in improvement in psychiatric symptoms, social functioning, quality of life, and treatment retention, as well as reducing the need for hospitalizations and crisis services, legal system involvement, self-harm, and substance abuse [10]. First-generation antipsychotics (FGAs) such as haloperidol have been proven to be effective; however, adverse effects such as Parkinsonism and tardive dyskinesia are a hindrance to long-term use [10]. Second-generation antipsychotics (SGAs) are thought to have equal or better efficacy than first-generation antipsychotics. SGAs are also more useful in the treatment of negative symptoms; however, SGAs can have harmful adverse effects on the cardiovascular (QT prolongation) and endocrine systems (hyperprolactinemia, which can cause sexual dysfunction, amenorrhea, galactorrhea and bone demineralization) [10]. An important aspect of treating a patient with schizophrenia is “patient-level characteristics” that can impact the outcomes of interventions [10].

## 2. Epidemiology

A meta-analysis found that the median estimate of the core incidence of schizophrenia is 15.2 per 100,000 [11]. The incidence rate ratio between males and females was found to be 1.4 [11]. The incidence ratio of schizophrenia varied significantly between migrants and native-born individuals, with the ratio being 4.6 migrant-to-native-born [11]. The incidence of urban and mixed urban-rural was found to be 19 and 13.3 per 100,000, respectively [11]. The median lifetime prevalence was 4.0 per 1000, and the lifetime morbid risk was 7.2 per 1000 [11].

## 3. Pathophysiology

It is hypothesized that schizophrenia occurs in individuals with a genetic predisposition to the disease, with environmental factors that over time lead to the progression of schizophrenia [12]. Adoption studies of individuals adopted within three days of birth show that the incidence of schizophrenia is the same in both the adopted subjects and those who stay with their biological family [13]. This suggests that environmental triggers in those with a genetic predisposition to schizophrenia occur sometime between conception and birth in the form of epigenetic changes are thought to be one of the main reasons for alter gene expression [14].

Serotonin plays an important role in cortical development and regulation in cognition, mood, and impulse control; as a result, it is hypothesized that abnormal serotonergic activity can contribute to the pathophysiology of schizophrenia [15,16]. Studies have shown that a reduction in the serotonin 2A receptor (HTR2A), as well as HTR7, is implicated in the pathogenesis of schizophrenia [17,18]. Neuroimaging data also suggest that levels of HTR2A may vary at different stages of the disorder [19]. Regulating the levels of HTR2A may also provide a new opportunity for treatment options [20].

Acetylcholine controls the cholinergic system in the central nervous system (CNS) and plays an important role in both learning and memory [21]. There are five different types of muscarinic receptors, all of which are differentially expressed throughout the CNS [22]. The variable roles of each type of muscarinic receptor and their respective distribution can help shed light on the pathophysiology of schizophrenia [20]. Low levels of the M1 receptor (CHRM1), in particular, have been implicated in the pathogenesis of schizophrenia [23,24]. The emergence of microarray technology has allowed researchers to track gene expression changes, including presynaptic functioning, G protein signaling, and myelination, all of which can have implications in both diagnosing and treating schizophrenia [20,25,26].

## 4. Risk Factors

The development of schizophrenia has been largely based on the neurodevelopmental theory [27]. The neurodevelopmental theory is based on a “single-hit” theory [28,29]. In the neurodevelopment theory, it is hypothesized that brain dysconnectivity is derived from deficits in dendritic spines during development arising from a combination of genetic factors and obstetric complications [30,31]. These deficits may progress further, leading to the expression of psychotic symptoms as a result of neuromaturation events such as aggressive synaptic pruning, or elevated cortisol leading to dendritic atrophy [28,32,33,34,35].

At present, it is thought that the pathogenesis of schizophrenia is not related to a “single hit,” rather it is the combination of “vulnerability factors” that alone have a weak individual effect but act together at critical points of neurodevelopment, which ultimately leads to schizophrenia [27]. These “vulnerability factors” include cannabis use, childhood trauma, and social defeat, among others [27]. Longitudinal studies suggest that there is up to a 40% greater risk of psychosis among people who have used cannabis [36,37]. It is thought that adolescents who use cannabis experience the onset of their first psychotic episode 2.7 years earlier than those with no history of cannabis use [38]. Childhood trauma including physical abuse, emotional abuse, sexual abuse, parental loss or divorce, parental substance abuse, poverty, and social defeat are thought to be vulnerability factors for the later development of schizophrenia [39]. Meta-analyses suggest that those with a history of childhood trauma have a risk of developing schizophrenia that is three times as high as those with no history of such trauma [40]. A history of childhood trauma also appears to be associated with worse positive symptoms as well as non-remission of positive symptoms [41]. Social defeat is defined as “losing a confrontation amongst any type of hostile dispute amongst humans” and consists of the stressors faced while being a part of a marginalized or excluded social or ethnic group [27]. Some studies suggest that the risk of schizophrenia is highest amongst immigrant groups who experience low social integration, poverty, alienation, and social uprooting [42].

## 5. Presentation

Schizophrenia presents a diverse combination of signs and symptoms, including distortions in perception, cognitive impairments, motor abnormalities, avolition and apathy, communication difficulties, and restricted affective expression, all of which are classified as being either positive, negative, cognitive, disorganized, mood or motor symptoms [43]. Positive symptoms include impaired reality testing with the presence of delusions, hallucinations, and reality distortions. Reality distortions indicate the formal onset of schizophrenia [43]. The positive symptoms of schizophrenia are likely a result of dopaminergic mesolimbic hyperactivity [44]. Negative symptoms involve the blunting of affect, which includes loss of motivation, poverty of speech, inability to experience pleasure, and lack of interest [45,46,47]. Negative symptoms can be further divided into primary and secondary negative symptoms [47,48]. Primary “deficit syndrome” negative symptoms are intrinsic to schizophrenia, while secondary negative symptoms are caused by extrinsic factors such as environmental deprivation and depression [43]. In contrast to the negative symptoms of schizophrenia, patients can also exhibit increased emotional reactivity [43]. The pathophysiology of the negative symptoms of schizophrenia is not well understood, and the symptoms are largely refractory to treatment [44,49,50]. Disorganized thought refers to the fragmentation of the logical, progressive, and goal-oriented nature of normal thought, which can range from mild circumstantiality to fully incoherent speech in the form of “word salad” [51]. Disorganized thought also includes derailment, clanging, and neologisms as well as poverty of thought [43]. Formal thought disorder often co-occurs with disorganized behavior [43]. Depression is present in the majority of schizophrenia patients, is more severe in those with substance abuse disorder, can occur in any phase of the disease, and significantly contributes to disease burden [52,53,54,55]. Along with positive and negative symptoms, schizophrenia also has cognitive impairment [56,57]. Cognitive impairments include impairments of episodic memory [58,59,60], processing speed [61], verbal fluency [62], attention [63,64], executive functions and working memory [65,66,67,68].

## 6. Current Treatment of Schizophrenia

There are many effective treatments available for schizophrenia, but patients experience repeated relapses related to nonadherence with oral medication formulations. The use of long-acting injectable (LAI) antipsychotics for patients who are unable to maintain a steady medication regimen is beneficial in reducing rehospitalization and treatment failure [69]. The current therapies available for schizophrenia are based on the dopamine hypothesis, which attributes positive symptoms to increased dopamine in the mesolimbic pathway and negative symptoms to decreased dopamine in the mesocortical pathway [70]. The dopamine D_2_ receptor is the primary target for drugs to alleviate psychotic symptoms, but it is associated with elevations in prolactin and extrapyramidal side effects. The currently used antipsychotic drugs fully or partially block the effects of dopamine at the D_2_ receptor [71]. Dopamine exerts its effects through G-protein coupled receptors that are further divided into the D_1_ subtype (D_1_ and D_5_ receptors) and D_2_ subtype (D_2_, D_3,_ and D_4_ receptors) [72,73].

### 6.1. First- and Second-Generation Antipsychotics

The first-generation antipsychotics block mostly D_2_ receptors in the brain and do not show any selectivity for the dopamine pathways present in the CNS, which leads to unwanted side effects associated with these antipsychotic drugs such as elevated prolactin and extrapyramidal symptoms [70]. First-generation drugs, such as haloperidol and chlorpromazine, have clinical efficacy in reducing the positive symptoms and decreasing the risk of relapse; however, they have little to no benefit for negative symptoms or the cognitive impairment associated with schizophrenia. Second-generation antipsychotics, such as clozapine and risperidone, are less likely to cause extrapyramidal symptoms (but both medications have other side effects that patients should be made aware of) and are efficacious in treating both positive symptoms and negative symptoms. Neither first nor second generation is superior at treating the cognitive deficits and achieves only moderate improvement in cognition when dosed properly [71]. Second-generation drugs and long-acting injectable formulations of antipsychotics have been associated with greater treatment efficacy. Specifically, long-acting injectables are more beneficial for patients with poor oral medication compliance. A cohort study conducted in 2017 by Tiihonen et al. determined that clozapine and long-acting injectable drugs like paliperidone are more effective at reducing the risk of treatment failure and rehospitalization than other antipsychotic drugs. Long-acting injectables have a 20–30% lower risk of rehospitalization compared with oral drugs [74].

### 6.2. Third-Generation Antipsychotics

While first- and second-generation antipsychotics are antagonists at the D_2_ receptor, third-generation antipsychotics are either partial D_2_ agonists or biased ligands. Third-generation antipsychotics such as aripiprazole, cariprazine, lumateperone, and brexpiprazole are the newest group of antipsychotics. Since third-generation drugs have been introduced, patients’ risk of experiencing parkinsonian symptoms, akathisia, and dystonia is substantially lower than with other antipsychotic agents. In general, patients that are only on third-generation drugs have had lower side effects.

There is still variability in patient response with any class of antipsychotic drugs. Some patients respond better to the older group of drugs, while others benefit more from a newer class of drugs, but this can be dependent on prescriber preference or comfort of use [70]. Even with the increasing number of antipsychotic drugs available to treat schizophrenia, management of the disease is far from optimal. Antipsychotic drugs have limitations that include patients lacking response to drug treatment, not controlling negative symptoms or cognitive defects, a wide range of debilitating side effects, and potential decreased survival due to pro-arrhythmic effects. Clozapine is the drug of choice for patients who are classified as “treatment-resistant” but has the potential to cause fatal agranulocytosis, as well as constipation (leading to bowel obstruction), severe cardiovascular issues and seizures. [70]. With the development of newer antipsychotics, the focus of treatment for schizophrenia has shifted from relieving the psychotic/positive symptoms to alleviating the negative symptoms and cognitive decline that affects the patient’s ability to recover and integrate back into society [75].

## 7. Aripiprazole Lauroxil Drug Information

### 7.1. Dosing Information

Aripiprazole lauroxil is an FDA-approved atypical antipsychotic indicated for the treatment of schizophrenia. Aripiprazole lauroxil can only be administered by intramuscular (IM) injection (deltoid 441 mg only or gluteal region 441 mg, 662 mg, 882 mg, 1064 mg) from a licensed healthcare professional. This method nullifies the need for daily oral administration and will help improve adherence to treatment in patients who are nonadherent with taking daily medications [76]. If a patient has never taken aripiprazole, oral tolerability must be established before aripiprazole lauroxil can be administered [77]. The half-life of oral aripiprazole is approximately 75 h, and it may take 2 weeks for some patients to reach tolerability status [78]. Oral dosage reflects how much injection dosage is given. If the patient were taking 10 mg per day, then they would receive a 441 mg injection every month. If the patient were taking 15 mg per day, then they would receive 662 mg every month, 882 mg every 6 weeks or 1064 mg every 2 months. If the patient were receiving 20 mg or higher, then they would receive a 882 mg injection every month. Oral aripiprazole should be administered for 21 consecutive days after the first aripiprazole lauroxil injection. The patient should be counseled on adverse reactions and side effects of the medication and counseled to go to the emergency room. Aripiprazole lauroxil can be given as a monthly dose of 441 mg, 662 mg, or 882 mg based on the needs of the patient. These doses are equal to 300 mg, 450 mg, and 600 mg of aripiprazole, respectively. All doses should be administered in the gluteal muscle except for the 441 mg dose, which can be given in the deltoid muscle dependent on patient preference [77,79].

### 7.2. Contraindications and Adverse Effects

Aripiprazole lauroxil is contraindicated in patients with a known hypersensitivity reaction to aripiprazole and is not approved for the treatment of elderly patients with dementia-related psychosis (boxed warning). Aripiprazole lauroxil is also associated with other adverse effects, including cerebrovascular accidents, neuroleptic malignant syndrome, tardive dyskinesia, hyperglycemia, dyslipidemia, orthostatic hypotension, leukopenia/neutropenia, seizures, cognitive and/or motor impairment, hyperthermia, and dysphagia [77]. Patients with a history of leukopenia or neutropenia should be monitored for the first few months of therapy and receive regular complete blood counts to ensure a normal white blood cell level. There is not sufficient data to determine the risks of birth defects or miscarriage in pregnant women using aripiprazole lauroxil, but withdrawal symptoms and extrapyramidal side effects have been observed in infants exposed to antipsychotics during the third trimester [80].

## 8. Mechanism of Action

Aripiprazole lauroxil is a prodrug of aripiprazole and is administered as an intramuscular injection. Once administered, aripiprazole lauroxil is first converted to N-hydroxymethyl aripiprazole by enzyme-mediated hydrolysis and is hydrolyzed again to aripiprazole [81,82]. Aripiprazole was originally reported to be a partial agonist at D_2_ and 5HT_1A_ receptors, with a combination of antagonistic activity at 5HT_2A_ receptors [77,82]. At present, it has been demonstrated that aripiprazole can act as an antagonist, partial agonist, or full agonist at D_2_ receptors [82]. Antagonistic activity at alpha_1_ receptors explains some of the adverse reactions that have been reported, such as orthostatic hypotension [83]. Contrary to other second-generation antipsychotics, aripiprazole displays a higher affinity for the dopamine receptor than the serotonin receptor. When the extracellular concentration of dopamine is high, such as in the mesolimbic circuit, aripiprazole can compete with dopamine as a partial antagonist. If the extracellular dopamine concentration is low, namely in dopamine circuits involved in cognition and working memory, aripiprazole can bind and partially activate other dopamine receptors. This unique mechanism of action gives aripiprazole the name “dopamine stabilizer” as it should ideally maintain dopamine levels in the tuberoinfundibular and nigrostriatal pathways to avoid hyperprolactinemia and extrapyramidal side effects [70,75]. Aripiprazole is also a partial agonist for D_2_ receptor-mediated recruitment of the β-arrestin-2 signaling pathway. This pathway appears to be critical in minimizing extrapyramidal side effects while maintaining antipsychotic efficacy [82]. Targeting the β-arrestin signaling pathways is promising in the design of future antipsychotic drugs [70].

## 9. Pharmacokinetics and Pharmacodynamics

### 9.1. Absorption and Distribution

After injection of aripiprazole lauroxil, aripiprazole is released into the systemic circulation after 5–6 days, and maximum concentration is reached approximately 41 days after administration. To bridge the gap created by slow absorption into the systemic circulation, patients should take oral aripiprazole for 21 days following the first injection [69]. An alternative regimen consisting of a nano-crystalline milled version of aripiprazole lauroxil and a 30 mg single dose of oral aripiprazole achieved the desired therapeutic dose in the same time frame as a 441 or 882 mg injection plus the 21-day oral initiation regimen [79]. Following absorption, aripiprazole displays broad extravascular distribution and has a volume of distribution of 268 L [83].

### 9.2. Metabolism

Once injected, aripiprazole lauroxil dissolves slowly and is cleaved by esterases into N-hydroxymethyl aripiprazole and lauric acid, which is a fatty acid found in human breast and cow’s milk. Through water-mediated hydrolysis, N-hydroxymethyl aripiprazole becomes aripiprazole and formaldehyde. The amount of formaldehyde formed by the metabolism of aripiprazole lauroxil is a minute amount compared with the amount produced by basic metabolism and a regular diet [69]. Aripiprazole lauroxil undergoes metabolism in the liver by CYP3A4 and CYP2D6 to dehydro-aripiprazole [69,83]. Pharmacokinetic differences may be present in individuals who are poor metabolizers of CYP2D6, and healthcare providers may need to adjust the dose based on patient response to treatment [80].

### 9.3. Elimination

The mean elimination half-life of aripiprazole lauroxil after administration of the final dose of 441 mg, 882 mg, and 1064 mg ranges from 53.9 to 57.2 days. Similar to the other long-acting injectables available, aripiprazole lauroxil has an extended pharmacokinetic profile due to an elimination rate that is faster than the absorption rate [84]. Thus, the calculated half-life for the injection of aripiprazole lauroxil is greater than the half-life of oral aripiprazole, which has a mean of 75 h [77]. After aripiprazole lauroxil is converted into its active metabolite, there is no measurable amount of prodrug present, making it unlikely that the rate of conversion or distribution of aripiprazole lauroxil is the cause of the slow elimination rate [84].

## 10. Clinical Studies: Safety and Efficacy

### 10.1. Phase I Studies

A randomized, open-label, single-dose study evaluated the bioavailability and safety of AL in adult patients with schizophrenia. Forty-six patients were randomized into two groups; 441-mg IM injection administered at the deltoid site or gluteal site. Although deltoid administration resulted in higher values for both areas under the plasma concentration-time curve from time zero to the last quantifiable plasma concentration (AUClast) and maximum plasma concentration (Cmax) of aripiprazole and dehydro-aripiprazole, the range of exposures overlapped for the two sites. Moreover, 38 of the 46 patients experienced at least one TEAE, which were all mild or moderately intense and did not lead to any discontinuations. The most common AE was injection-site pain, which occurred at a higher frequency with deltoid administration (63.6%) than gluteal administration (better absorption) (27.3%) [85].

A 44-week, open-label study evaluated the pharmacokinetics and safety of 1064-mg AL in patients with schizophrenia by randomizing 105 patients to one of three groups; 1064-mg dosed every 8 weeks, 882-mg dosed every 6 weeks, and 441-mg dosed every 4 weeks for a total of 4, 5 and 7 injections. Overall, 1064-mg AL provided similar mean aripiprazole concentrations to 882-mg AL, with both resulting in higher aripiprazole exposure than 442-mg AL. Injection-site pain was the most frequent AE in all three regimens, with the overall incidence of injection-site reactions of 11.5%. Although most AEs were mild or moderate, severe AEs (SAEs) occurred in all 3 groups but, with the exception of one case of increased psychosis, were considered unrelated to AL. The incidence of discontinuation due to TEAEs was 2.9%, 11.8% and 5.7% in the 1064-mg, 882-mg and 441-mg groups, respectively. The incidence of AEs associated extrapyramidal symptoms (EPS) was the highest in the 882-mg group [86].

### 10.2. Phase III Studies

A 12-week, randomized, double-blind, placebo-controlled study (RDBPC) demonstrated the safety and efficacy of two doses of Aripiprazole Lauroxil (AL) in treating patients with schizophrenia experiencing an acute relapse. Patients were randomized to AL 441-mg, AL 882-mg or placebo, with a dosing regimen of every 4 weeks, administered in the gluteal muscle. Outcomes studied were an improvement from baseline to day 85 in Positive and Negative Syndrome Scale (PANSS) total score and the Clinical Global Impressions-Improvement scale (CGI-I) score at day 85 for AL groups compared with placebo. The placebo-adjusted least-squares mean differences of PANSS total score for AL 441-mg and AL 882-mg were −10.9 (1.8) (*p* < 0.001) and −11.9 (1.8) (*p* < 0.001). The CGI-I scores for both AL groups were also significantly better than the placebo (Wilcoxon rank sum test: *p* < 0.001). The incidence of severe TEAEs was similar in all three groups, with insomnia, akathisia, headache, and anxiety common TEAEs. Akathisia was observed to have an incidence in the AL groups twice that of the placebo group, the only TEAE to reach this. The overall incidence of injection-site reactions was low, and pain was the most common injection-site reaction. The rate of discontinuation due to AEs was 6.8%, 2.9%, and 17.9% for the 441-mg, 882-mg, and placebo groups, respectively [87].

Nasrallah et al. carried out a 52-week, open-label extension study of the 12-week RDBPC [87] to assess changes in endocrine and metabolic parameters due to AL monotherapy in outpatients with schizophrenia. For the whole population, there was a decrease in serum prolactin levels, slight decreases in levels of total cholesterol and triglycerides, and slight increases in fasting blood glucose, HbA1c, and body weight. No clinically meaningful difference was found for these parameters between the two treatment arms (AL 441-mg and 882-mg). The incidence of TEAEs was similar in both groups, with insomnia, increased weight and anxiety TEAEs. The rate of discontinuation due to AEs as well as the incidence of SAEs were both higher in the 882-mg group [88].

### 10.3. Phase IV Studies

A prospective, 6-month, open-label study evaluated clinical outcomes and the safety of switching to AL from paliperidone palmitate (PP) and risperidone long-acting injection (RLAI) in 51 patients with schizophrenia who did not tolerate the latter or did not see improvement in symptoms. Both Clinical Global Impressions–Severity (CGI-S) and Brief Psychiatric Rating Scale (BPRS) scores showed statistically significant improvements at the end of treatment period and after 6 months, respectively. Moreover, the overall retention rate was 68.6%, and the incidence of discontinuation due to medication-related reasons was <10%. The incidence of any AE was 41.2%, any SAE was 9.8%, and of drug-related AEs was 17.6%. Common AEs included psychotic disorder (7.8%), anxiety (5.9%) and suicidal ideation (5.9%) [89].

### 10.4. Other Studies

A series of post-hoc analyses of the phase 3, 12-week RDBPC [87] were performed and are described below.

Potkin et al. carried out a subgroup analysis to evaluate the efficacy and safety of AL treatment for patients who had severe psychotic symptoms (defined as having a PANSS total score greater than the median score of 92). A mixed model for repeated measures (MMRM) showed statistically significant decreases in placebo-adjusted mean change in PANSS total score and PANSS Positive, Negative, and General Psychopathology scores for both AL 441 mg and AL 882 mg. The overall responder rates for both AL doses were also significantly higher compared with placebo: 49% (*p* < 0.001) and 61% (*p* < 0.001), respectively. Finally, effect sizes for this subgroup of patients were higher for both doses than for the group of patients with less severe symptoms (defined as PANSS ≤ 92). Moreover, AL-882 mg resulted in a higher responder rate and also produced a higher effect size in the more severely ill patient subgroup than the AL 441-mg. Akathisia, headache, insomnia, and anxiety were among common AEs in the patient subgroup analyzed [90].

Targum et al. analyzed the effect of age and gender on the treatment response of AL versus placebo. For the analysis, patients were stratified into the following age groups: <30, 30–39, 40–49, and 50–69 years. ANCOVA analysis showed no significant interaction effects between age and treatment for both AL 441 mg versus placebo and AL 882 mg versus placebo; (F = 0.27; *p* = 0.60) and (F = 0.92; *p* = 0.34), respectively. Odds ratios (ORs) for treatment response rates, (response defined as ≥30% total PANSS score improvement from baseline) on day 85 also showed higher association for either AL dose versus placebo for all age groups. No interaction effect between gender and treatment was observed, with outcomes for both men and women being better in both AL groups than placebo [91].

Correll et al. assessed social and functional outcomes using 6- and 4-item Positive and Negative Syndrome Scale (PANSS) Prosocial subscales and Personal and Social Performance (PSP) total score. Statistically significant improvements were observed in both 6- and 4-item PANSS Prosocial Scores for both doses of AL versus placebo, with the treatment effect sizes with AL 441 mg versus placebo being Cohen’s d = 0.52 and AL 882 mg versus placebo being Cohen’s d = 0.49. PSP total score results also showed a similar trend, with a statistically significant increase for both doses versus placebo, with treatment effect sizes of Cohen’s d = 0.51 and 0.59 for AL 441-mg or AL 882-mg compared with placebo, respectively [92].

Citrome et al. analyzed the effectiveness of AL using the number needed to harm (NNH) and the number needed to treat (NNT). Pooled AL results (using both AL 441-mg and 882-mg results) produced NNT values of 4, 6, 10 and 26 when the response thresholds were ≥20%, ≥30%, ≥40%, and ≥50% decrease in PANSS total score from baseline to day 85, respectively. A Cohen’s d of 0.61 (95% CI: 0.44–0.79) for the PANSS total score change and an NNH of −8 (95% CI: −6 to −15) for discontinuation due to AEs were both calculated for pooled doses of AL versus placebo. Moreover, some AEs produced a statistically significant estimate of double-digit NNH values for pooled AL doses versus placebo, including akathisia, toothache, increased blood creatine phosphokinase, and increased weight [93].

Nasrallah et al. analyzed the effect of AL on metabolic and endocrine parameters such as body weight, glycemic control, and lipid and prolactin levels. At day 85, in both AL groups relative to placebo, mean body weight increased slightly while prolactin levels decreased in both men and women. Changes in lipid parameters (total cholesterol, LDL cholesterol, and triglycerides), plasma glucose, and HbA1c were insignificant. The overall incidence of AEs related to metabolic effects was low and was 2.4%, 1.4%, and 2.4% in AL 441 mg, AL 882 mg, and placebo groups, respectively [94].

Citrome et al. also analyzed the effect of AL on agitation and hostility levels of patients with schizophrenia using three outcomes: PSP disturbing and aggressive behavior domain, PANSS Hostility item (P7) score in the subpopulation with a PANSS Hostility item P7 score of more than 1 at baseline, and the PANSS excited component (PANSS-EC) score. Significant improvements occurred for all three outcomes: (*p* < 0.05) for reduction in PANSS Hostility item P7 score for the subpopulation treated with 882-mg, *p* < 0.001 for mean change in PANSS-EC scores for both groups versus placebo, and *p* = 0.007 and *p* < 0.001 for reduction of incidence of PSP disturbing and aggressive behavior domain for AL 441 mg vs. placebo and AL 882 mg vs. placebo, respectively. Additionally, greater benefits with AL 882-mg were indicated because it showed a significant reduction in the mean PANSS Hostility item P7 score in the subpopulation, even after controlling for positive symptoms and somnolence or akathisia, thus producing a specific anti-hostility effect, and also showed greater improvement than AL 441mg group for PANSS-EC [95].

McEvoy et al. carried out an analysis of the 52-week extension study [88] to evaluate the durability of long-term AL treatment in a subgroup population (patients who had completed both the initial 12-week study and the subsequent 52-week study and were randomized to either the AL 441-mg group or 882-mg group in the extension study). For both AL groups, a statistically significant decrease occurred for both PANSS total score and CGI-S scores from week 12 to week 64. By week 64, remission rates were 73.8% and 68.1% in the 441 mg and 882 mg groups, respectively, with the median remission time from the beginning of the 12 weeks being 16.1 and 16.4 weeks, respectively. Rates of treatment discontinuation were low for the 441 mg (27.25) and 882 mg (34%) groups in the extension study, with withdrawal due to adverse events being 2.5% and 5%, respectively [96].

Moreover, a targeted systematic review of 31 RCTs (7 primary studies and 24 post-hoc analyses) compared LAIs to placebo, oral antipsychotics (OAPs) and other LAIs. AL was among the LAIs included. Results showed that LAIs were wholly superior to placebo and partly superior to OAPs regarding prevention of relapse and hospitalization, with no observed differences in comparison to other LAIs. LAIs were comparable to OAPs in other domains such as all-cause discontinuation, functioning, quality of life, and tolerability and were associated with higher patient satisfaction and service engagement. Moreover, although results from recent meta-analyses were inconclusive, they did not show an advantage for OAPs over LAIs [97]. Table 1 summarizes the studies discussed in this section.

### 10.5. Comparison Study

A network meta-analysis (NMA) carried out an indirect comparison between AL and paliperidone palmitate (PP) using data from four Phase III clinical trials. Results demonstrated that all four effective-treatment arms (AL 441 mg and 882 mg; and PP 156 mg and 234 mg) showed effectiveness by reductions in PANSS total score versus placebo. The extent of these reductions was similar, with the range of mean differences spanning from −8.12 to −12.01, with overlapping 95% credible intervals. These results, in addition to a similar incidence of TEAEs for both AL and PP, supported the finding that AL and PP were equally efficacious [98].

A study evaluated the differences in the steady-state aripiprazole plasma concentrations of two long-acting injectable (LAI) formulations of aripiprazole: aripiprazole once-monthly 400 mg (AOM 400) and AL. The outcomes measured were the median minimum plasma concentration at steady state (Cmin,ss), calculated based on observations from previous clinical trials, and the average plasma concentration at steady state (Cavg,ss), based on simulations. Cavg,ss values for AOM 400 and AL 882 mg administered every 4 weeks were comparable, while AL 441 mg resulted in considerably lower Cavg,ss values. Cmin,ss values for the intragluteal injection of AOM 400 and the intragluteal injection of AL 882 mg every 4 weeks were also found to be similar (200 ng/mL and 175 ng/mL, respectively) [99]. Table 2 summarizes the comparative studies discussed here.

**Table 1:** Clinical efficacy and safety.

Author (Year)	Groups Studied and Intervention	Results and Findings	Conclusions
Turncliff R. et al. (2014) [85]	A phase 1, randomized, open-label, single-dose study. 46 patients were randomized to two groups; 441 mg IM injection administered at deltoid site or 441 mg IM injection at gluteal site. Maximum plasma concentration (C_max_) and area under the plasma concentration time curve from time zero to infinity (AUC_0–inf_) of aripiprazole and its metabolite dehydro-aripiprazole were measured. AEs were measured as tolerability outcomes	Deltoid administration resulted in higher C_max_ aripiprazole concentration, while AUC_0–inf_ was similar for both sites. Dehydro-aripiprazole exposure was 33% and 36% of aripiprazole exposure for deltoid and gluteal administration, respectively. The most common AE was injection site pain, with a higher incidence in the deltoid group.	Deltoid and gluteal injection sites provided similar levels of exposure and were both well-tolerated in patients with chronic stable schizophrenia.
Meltzer H. et al. (2015) [87]	A phase 3, randomized, double-blind, placebo-controlled trial. 623 patients (ages 18–70) experiencing an acute exacerbation of schizophrenia were randomized 2:2:1:1 to AL 441 mg, AL 882 mg, placebo low volume, and placebo high volume, respectively. Injections were administered in the gluteal muscle on days 1, 29 and 57. The primary efficacy endpoint was change from baseline to day 85 in PANSS total score, and the secondary efficacy endpoint was CGI-I score at day 85.	Placebo-adjusted least squares mean differences of PANSS total score were significantly lower for both AL 441 mg and AL 882 mg; *p* < 0.001 for both. CGI-I scores for both AL groups were also significantly better than placebo (Wilcoxon rank sum test: *p* < 0.001).	Both doses of AL were highly efficacious in treating an acute exacerbation of schizophrenia, with a safety and tolerability profile similar to oral aripiprazole, therefore representing a new treatment option.
Nasrallah H. et al. (2016) [94]	Patients with schizophrenia were randomly assigned to AL 441 mg, AL 882 mg, or placebo intramuscularly. Body weight, body mass index, fasting blood glucose and serum lipids, glycosylated hemoglobin (HbA1c), and prolactin were the parameters evaluated for 12 weeks. Treatment-emergent adverse events (AEs) were used for safety evaluations.	For both AL groups compared with placebo, mean body weight increased slightly and prolactin levels decreased, while changes in lipid parameters (total cholesterol, LDL cholesterol and triglycerides), plasma glucose, and HbA1c were insignificant. The incidence of AEs related to metabolic parameters was low.	Both AL 441 mg and AL 882 mg showed a small weight gain compared with placebo but otherwise reflected a low-risk metabolic profile.
Citrome L. et al. (2016) [95]	Patients with schizophrenia were randomly assigned to AL 441 mg, AL 882 mg, or placebo intramuscularly. Study endpoints were PANSS Hostility item (P7) score in the subpopulation of patients with a PANSS Hostility item P7 score of more than 1 at baseline, PSP disturbing and aggressive behavior domain and PANSS excited component (PANSS-EC) score.	AL groups compared with placebo resulted in significant lowering in proportion with PANSS Hostility item P7 more than 1 at endpoint (*p* < 0.05), as well as significant improvement (*p* < 0.05) in PANSS excited component score. The proportion of patients with aggressive behavior on the Personal and Social Performance scale was also significantly lower for both 441 mg and 882 mg compared with placebo (*p* = 0.006 and *p* < 0.001, respectively).	AL treatment displayed efficacy in reducing agitation and hostility in patients with schizophrenia.
Nasrallah H. et al. (2017) [88]	Phase 3, international, 52-week, open-label extension study with 478 patients. 368 received AL 882 mg and 110 received AL 441 mg as their fixed-dose regimen. Of the 478, 236 entered from a phase 3 study and 242 entered as outpatients with no prior AL exposure and a Clinical Global Impression–Severity score of ≤3 [mild] at screening. Metabolic parameters of weight, fasting blood sugar, lipids and serum prolactin were assessed for changes	The mean changes from baseline in the overall population were +1.1 mg/dL for glucose, +0.07 for glycated hemoglobin (HbAlc), −3.3 mg/dL for total cholesterol, and −5.3 mg/dL for triglycerides, while prolactin change from baseline was −8.7 ng/mL (14.7) for men and −14.9 (43.4) ng/mL for women. The retention rates at 6 months and 1 year were 86% and 68%, respectively.	Long-term AL treatment lead to slight lowering of serum with insubstantial changes in other assessed parameters.
Potkin S. et al. (2017) [90]	Patients with schizophrenia were randomly assigned to AL 441 mg, AL 882 mg, or placebo intramuscularly. Post-hoc analysis of the patient subgroup with severe psychotic symptoms (PANSS total score greater than the median score of 92). Primary outcome measured was mean change from baseline to day 85 in PANSS Total score. Categorical responder rate (defined as ≥30% improvement in PANSS Total score or a final CGI-I score of ≤2 [very much or much improved]) was also evaluated.	Both AL 441 mg and 882 mg demonstrated statistically significant and clinically meaningful improvements in PANSS Total score, with placebo-adjusted differences of −14.7 (*p* < 0.0001) and −16.6 (*p* < 0.0001), respectively, as well as significant findings with responder rates (*p* < 0.001) for both groups vs. placebo. Moreover, AL-882 mg resulted in a higher responder rate.	Both doses of AL demonstrated robust efficacy in treating patients with severe psychotic symptoms. The 882 mg dose displayed a numerically greater improvement in symptoms and proportion of responders.
Targum S. et al. (2017) [91]	Patients with schizophrenia were randomly assigned AL 441 mg, AL 882 mg, or placebo intramuscularly. Post-hoc gender and age analysis of AL treatment response, with age groups <30, 30–39, 40–49, and 50–69 years old. The primary outcome measured was change in total PANSS score from baseline to day 85. Categorical treatment response (defined as ≥30% total PANSS score improvement from baseline) was also measured.	ANCOVA analysis of change in PANSS score showed no significant interaction effects between age and treatment for both AL 441 mg versus placebo and AL 882 mg versus placebo; (F = 0.27; *p* = 0.60) and (F = 0.92; *p* = 0.34), respectively. The odds ratios (ORs) for treatment response rates showed higher association for either AL dose versus placebo for all age groups. No interaction effect between gender and treatment was observed, with outcomes for both men and women being better in AL groups than placebo.	AL 441 mg and AL 882 mg led to significant improvement in mean total PANSS score and categorical treatment response compared with placebo, regardless of patient age and gender.
McEvoy J. et al. (2017) [96]	Post-hoc analysis of long-term outcomes in patients who had completed both a phase 3 and extension study. Outcomes measured were rates of retention and remission, as well as treatment response trajectories, as measured by PANSS total and CGI-S item scores in AL 441 mg and AL 882 mg groups.	A statistically significant decrease occurred for PANSS total score (*p* < 0.0001 for both groups) and CGI-S scores ((*p* < 0.0001 for both groups). By week 64, remission rates were 73.8% and 68.1% in the 441 mg and 882 mg groups, respectively, with the median remission time from the beginning of the 12 weeks being 16.1 and 16.4 weeks, respectively. Retention rates were 72.8% and 66.0% for the 441 mg and 882 mg groups, respectively.	Both Al 441 mg and AL 882 mg exhibited continued therapeutic benefit in the long-term, as evidenced by high retention rates and significant improvements in clinical symptoms.
Miller B. et al. (2019) [89]	A phase 4, 6-month, prospective, open-label study in which 51 patients switched from either PP or RLAI to AL. Outcomes measured were CGI-S scores, BPRS scores, all-cause and medication-related discontinuation and adverse events. AEs were also assessed.	CGI-S and BPRS scores showed significant improvement, and the retention rate with all-cause and medication-related discontinuation rates at the end of 6 months was 30% and 9%, respectively. The retention rate at the end of 6 months was 68.6%, while the incidence of AEs was 41.2%, with the most frequent psychotic disorder, anxiety and schizophrenia.	Patients being treated with PP or RLAI who experience continued symptoms or tolerability issues can switch to AL, as the latter is well-tolerated.
Correll C. et al. (2019) [92]	Patients with schizophrenia were randomly assigned to AL 441 mg, AL 882 mg, or placebo intramuscularly. Post-hoc social and functional outcomes analysis of 596 of the total 623 patients. Outcomes measured were 6-item PANSS Prosocial subscale, 4-item PANSS Prosocial subscale and Personal and Social Performance (PSP) total score.	Both 6- and 4-item PANSS Prosocial Scores showed significant improvement for both doses of AL versus placebo, with the treatment effect sizes for PANSS Prosocial scores with AL 441 mg versus placebo being Cohen’s d=0.52 and AL 882 mg versus placebo being Cohen’s d=0.49. There was a significant increase in PSP total score for both doses versus placebo, with treatment effect sizes of Cohen’s d = 0.51 and 0.59 for AL 441-mg or AL 882-mg compared with placebo, respectively.	Treatment with AL 441 mg and AL 882 mg compared with placebo produces significant improvements in social functioning.
Citrome L. et al. (2019) [93]	Patients with schizophrenia were randomly assigned to AL 441 mg, AL 882 mg, or placebo intramuscularly. Post-hoc analysis with categorical efficacy and tolerability. Outcomes measured were number needed to treat (NNT) for therapeutic outcomes, number needed to harm (NNH) for adverse outcomes, and likelihood to be helped or harmed (LHH).	For pooled doses of AL, an NNT of 6 (95% CI: 5–11) was calculated when the response threshold was ≥30% improvement from baseline PANSS total score. For discontinuation due to AEs, a NNH estimate of −8 (95% CI: −6 to −15) for the pooled doses of AL vs placebo was calculated, while for Akathisia, the NNH was 14 (95% CI: 9–33). LHH value, using the NNT for response (≥30% reduction from baseline in PANSS total score) and the NNH for akathisia, was 2.3.	Aripiprazole lauroxil is efficacious and well-tolerated in the treatment of an acute exacerbation of schizophrenia, as evidenced by NNT and NNH values.
Peters L. et al. (2019) [97]	A systematic review of 31 RCTs (7 primary studies and 24 post hoc analyses) to evaluate comparisons of LAIs to placebo, OAPs or another LAI and 5 meta-analyses of RCTs comparing LAI to OAPs, all published 2016–2019. AL was among the LAIs analyzed.	LAIs were vastly superior to placebo and partly superior to OAPs for prevention of relapse and hospitalization and were comparable to OAPs for all-cause discontinuation, functioning, quality of life, and tolerability, as well as being associated with higher patient satisfaction and service engagement. Results from recent meta-analyses did not show an advantage for OAPs over LAIs.	Results from RCTs show that LAIs outperform placebo but are better than OAPs in only some aspects. Meta-analyses results do not reveal an advantage for OAP vs. placebo.
Weiden P. et al. (2020) [86]	A phase I, open-label, multicenter study, with 105 patients randomized to one of three AL dose regimens of 1064-mg injections every 8 weeks, 882-mg injections every 6 weeks, or 441-mg injections every 4 weeks, for a total of 24 weeks, with a 20-week follow-up. Plasma aripiprazole concentrations and AEs were evaluated.	Both 1064-mg and 882-mg regimens provided comparable aripiprazole exposure, higher than that of 441-mg. Most AEs were mild or moderate, with injection-site pain being the most frequent AE in all three regimens. Discontinuation due to TEAEs was 2.9%, 11.8% and 5.7% in the 1064-mg, 882-mg and 441-mg groups, respectively.	All three dosing regimens provide continuous exposure to AL, including 1064-mg administered every 2 months, and all produced a safety profile similar to previously established safety profiles.

## 11. Conclusions

Schizophrenia consists of positive symptoms, negative symptoms, and cognitive dysfunction. Several theories regarding pathogenesis have been proposed, including the neurodevelopmental theory and dopaminergic imbalance. There are several risk factors that contribute to the development of schizophrenia, including genetic factors, history of cannabis use, and childhood trauma. The pharmacological treatment of schizophrenia is largely based on mitigating dopaminergic imbalance.

First-generation antipsychotics work by non-selectively blocking D_2_ receptors in the CNS. While effective at mitigating positive symptoms, FGAs lead to unwanted extrapyramidal symptoms and elevated prolactin levels due to non-selectivity. FGAs have not been shown to reduce negative symptoms or improve cognitive impairment. Second generation antipsychotics also work by blocking dopaminergic receptors. SGAs have been shown to produce less extrapyramidal symptoms and can treat both positive and negative symptoms; however, SGAs show no improvement in cognitive dysfunction. Third generation antipsychotics, including aripiprazole, can be characterized as D_2_ partial agonists or D_2_-biased ligands.

Aripiprazole can act as an antagonist in areas with high dopamine levels and an agonist in areas with low dopamine levels, thus giving it the name “dopamine stabilizer.” The use of aripiprazole has also been shown to minimize extrapyramidal symptoms when compared with first- and second-generation antipsychotics. Aripiprazole lauroxil is a prodrug of aripiprazole and is given as an IM injection, which helps improve treatment rates in patients who are not compliant in taking once-daily oral medication. AL is metabolized by CYP3A4 and CYP2D6 in the liver. The use of AL is contraindicated in patients with known aripiprazole hypersensitivity and in dementia-related psychosis. Side effects are known to include weight gain, akathisia, neuroleptic malignant syndrome, tardive dyskinesia, orthostatic hypotension due to alpha_1_ blockade, leukopenia, and neutropenia. The use of AL has shown significant statistical and clinical efficacy in treating the symptoms of schizophrenia.

## Figures and Tables

**Table 2:** Comparative studies.

Author (Year)	Groups Studied and Intervention	Results and Findings	Conclusions
Cameron C. et al. (2017) [98]	A NMA, with searches from MED- LINE, Embase, Cochrane CENTRAL, PsycINFO, ClinicalTrials.gov, International Clinical Trials Registry Platform, and gray literature which identified four Phase III clinical trials (1 of AL and 3 of PP), for indirect comparison of AL and PP. Active-treatment groups were AL (441 mg and 882 mg monthly) and PP (156 mg and 234 mg monthly).	All four groups versus placebo resulted in substantial reduction in acute symptoms, as measured by the positive and negative syndrome scale; the range of mean difference was −8.12 to −12.01, with overlapping 95% credible intervals. No differences in efficacy, safety or tolerability were found between active-treatment groups.	AL and PP are equally efficacious in treating adults with acute exacerbation of schizophrenia.
Salzman P. et al. (2017) [99]	Aripiprazole steady-state plasma concentrations for two different LAI formulations, (aripiprazole once-monthly 400 mg [AOM 400] and AL), were compared. C_avg,ss_ for AOM 400 were obtained from a population Pk model, while those for AL were obtained from the Center for Drug Evaluation and Research (CDER). C_min,ss_ for both LAIs were obtained from previously published clinical trials.	C_avg,ss_ values for AOM 400 and AL 882 mg given every 4 weeks were comparable, while AL 441 mg resulted in considerably lower C_avg,ss_ values. C_min,ss_ values for the intragluteal injection of AOM 400 and the intragluteal injection of AL 882 mg every 4 weeks were also similar (200 ng/mL and 175 ng/mL, respectively).	Both AOM 400 mg and AL 882 mg administered every 4 weeks provided similar Aripiprazole steady-state plasma concentrations.

## Data Availability

No data was collected in this manuscript but all were cited as appropriate and can be found in the reference section.
